# Ultra-Wideband (UWB) Antenna Sensor Based Microwave Breast Imaging: A Review

**DOI:** 10.3390/s18092951

**Published:** 2018-09-05

**Authors:** Md. Zulfiker Mahmud, Mohammad Tariqul Islam, Norbahiah Misran, Ali F. Almutairi, Mengu Cho

**Affiliations:** 1 Department of AIS, Jagannath University, Dhaka 1100, Bangladesh; zulfikerm@siswa.ukm.edu.my; 2 Centre of Advanced Electronic and Communication Engineering, Universiti Kebangsaan Malaysia, 43600 Bangi, Malaysia; bahiah@ukm.edu.my; 3 Laboratory of Spacecraft Environment Interaction Engineering (LaSEINE), Kyushu Institute of Technology, Kitakyushu 804-8550, Japan; cho@ele.kyutech.ac.jp; 4 Electrical Engineering Department, Kuwait University, Kuwait City 13060, Kuwait; ali.almut@ku.edu.kw

**Keywords:** antenna sensor, microwave imaging, breast tumor, ultra-wideband (UWB), dielectric properties, breast phantom, high gain

## Abstract

Globally, breast cancer is reported as a primary cause of death in women. More than 1.8 million new breast cancer cases are diagnosed every year. Because of the current limitations on clinical imaging, researchers are motivated to investigate complementary tools and alternatives to available techniques for detecting breast cancer in earlier stages. This article presents a review of concepts and electromagnetic techniques for microwave breast imaging. More specifically, this work reviews ultra-wideband (UWB) antenna sensors and their current applications in medical imaging, leading to breast imaging. We review the use of UWB sensor based microwave energy in various imaging applications for breast tumor related diseases, tumor detection, and breast tumor detection. In microwave imaging, the back-scattered signals radiating by sensors from a human body are analyzed for changes in the electrical properties of tissues. Tumorous cells exhibit higher dielectric constants because of their high water content. The goal of this article is to provide microwave researchers with in-depth information on electromagnetic techniques for microwave imaging sensors and describe recent developments in these techniques.

## 1. Introduction

Breast cancer has become the most threatening disease to women. According to projections, more than 24 million new breast cancer cases will be diagnosed by 2035. According to the National Cancer Registry of Malaysia (NCR), one in 19 Malaysian women will be diagnosed with breast cancer by the age of 85. Approximately 4000 cases occur each year, mostly in women between 35 and 60 years, with 40% of cases occurring in women younger than 50 [1,2]. An increasing population and longer life spans have contributed to the rise of cancer. Breast cancer occurs because of the presence of malignant cells inside breast tissue [3]. At present, the most common killer of urban women is breast cancer, which has become a significant global health problem. Breast cancer is not incurable. From previous research, we know that a key factor in curing breast cancer is reliably diagnosing it at an early stage. With early breast cancer detection and treatment, the survival rate can reach 97%. This underscores the urgent need for reliable and highly efficient early breast cancer detection methods [4].

The basic principle of microwave imaging is to send microwave signals into human tissue [5] and analyze changes in the back-scattered signal, which reflect differences in the electrical properties of tissues. The remarkable variations in the back-scattered signal can be used to identify unwanted tumor cells inside the breast, which exhibit higher dielectric constants than normal breast tissues. 

Antenna sensors play a key role in microwave imaging systems (MISs). In an MIS, the antenna acts as a transmitting and receiving sensor. The transmitting antenna sends microwave signals through the breast, whereas scattered signals from the breast tissue are collected by the receiving antenna. Recent studies that use the antenna as a sensor in an MIS have indicated that the antenna should have the following properties [6,7,8]: high gain and small size; directive radiation of power; the ability to transmit a wide range of frequencies with higher efficiency; model simplicity; compatible penetration of human tissue; and the ability to operate at both low and high frequencies. 

Emerging ultra-wideband (UWB) antenna technology has some unique features, like high-speed data rates, very small interference, simple low-cost designs, low power spectrum density, robust multipath applications, and higher precision ranges. Since 2002, the Federal Communications Commissions (FCC) has allowed UWB bands of 7.5 GHz (from 3.1 to 10.6 GHz) for commercial usage [9]. UWB has a wide range of applications in high speed communications, like radars, short-distance applications like PC peripherals, wireless local area networks (WLANs), and microwave imaging used to scan the human body. 

In this paper, we discuss the limitations of current detection techniques, and describe proposed microwave sensor imaging techniques, imaging algorithms, numerical analysis methods, and physical quantifications. Additionally, we describe a few relevant clinical trials.

## 2. Limitations of Current Detection Techniques

Over the past few decades, several clinical imaging technologies have been developed that can produce valuable interior pictures of the human body. X-ray mammography, computed tomography (CT), ultrasounds (US), and magnetic resonance imaging (MRI) are frequently used diagnostic tools for detecting breast cancer [10,11]. However, X-ray mammography produces a relatively high number of both false negative diagnoses (between 10% and 30%) and false positive diagnoses (more than 5%). Additionally, mammography uses radiation that requires uncomfortable compression of the breast during the examination and is of limited value for younger women. Moreover, it is evident that the ionization caused by X-ray mammography [10] represents a severe health threat and there is even a chance of ionization causing the canceration of healthy tissue. US is an alternative detection method with a 17% false-negative rate. Additionally, deep-lying or solid cancerous tissues are difficult to detect.

MRIs can produce high-resolution images but is costly and time consuming. To improve the detection system, combinations of different models have also been investigated. To achieve this, a new, efficient, nonionizing, low cost, portable, and comfortable approach is in high demand as a complementary tool to current technology [12].

In [13], a total of 258 patients were studied, where 177 had malignant tumors and 177 had benign tumors. The sensitivity (ratio of detected malignant tumors to the total number of patients with malignant tumors), specificity (ratio of detected benign tumors to the total number of patients with benign tumors), positive predictive value (ratio of correctly detected positive malignant tumors to total positive diagnoses), and accuracy (ratio of total patients with diagnosed benign or malignant tumors to total patients) were studied to test the present detection techniques. A performance comparison of the different detection techniques investigated in [13,14] is presented in Table 1.

Table 1 shows that the percentage of correct diagnoses is limited. The highest sensitivity (94.4%) was obtained by combining mammography, MRI, and some clinical approaches. The maximum accuracy obtained was 75.6%, which indicates that approximately one out of every four diagnoses is false. 

Microwave imaging system (MIS) methods have become hot topics of investigation both as complementary tools and alternatives to available techniques. MIS can overcome the disadvantages of other methods such as false indications, low-resolution scans, higher cost, and patient discomfort. MIS is advantageous because of its high positive rate, low cost, comfort, high data rate, low complexity, portability, and low spectral power density.

## 3. UWB Sensor Based Microwave Imaging

**Figure 1:**
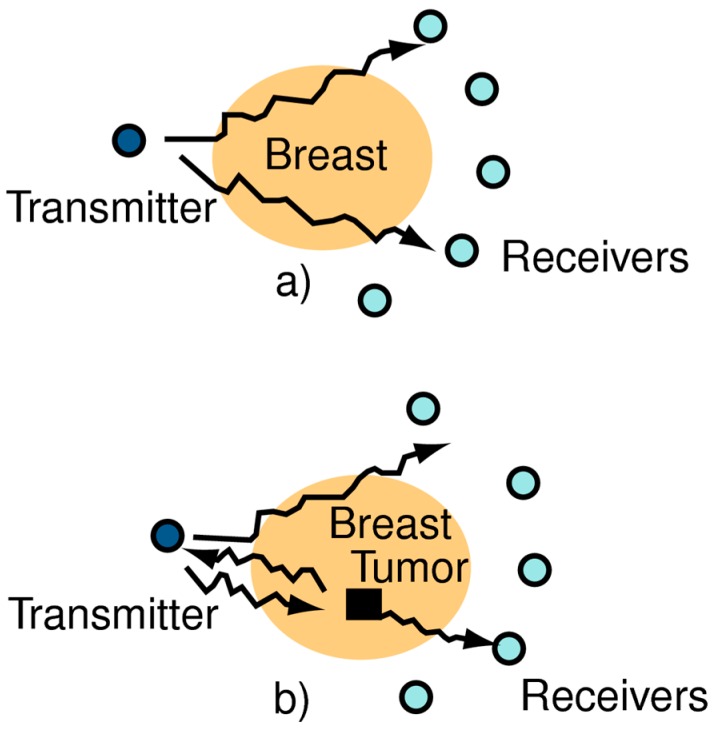
Basic microwave imaging problem (**a**) Reflected waves from breast without a tumor (**b**) Reflected waves from breast with a tumor. Changes indicate differences from tumor-free breast [15].

Microwave imaging is defined by Fear et al. in [15] as “seeing the internal structure of an object by means of electromagnetic fields at microwave frequencies of 300 MHz to 30 GHz”. The basic principle of the technology is that microwaves travel from the transmitter through the breast and are detected by a receiver located on the opposite side. A change occurs in the waves traveling through the breast if they pass through a tumor; in such cases, the incident wave is scattered. This strongly affects the amount of incident wave energy at the receiver, as shown in Figure 1. In microwave imaging, antennas are used as transceivers. Typically, two different types of antennas are used in MISs: (1) resonance type antennas; and (2) antennas designed based on high-profile traveling wave principle, like Vivaldi antennas. A UWB antenna can operate in both low- and high-frequency ranges, with unique features like non-contact remote operations, intrinsic electrical transducers, environmental friendliness, biocompatibility, and biological friendliness [16]. These features have been of interest to researchers because of their advantages for medical applications. Researchers have proposed using several types of UWB antennas for microwave imaging applications: omnidirectional vs. directional radiation patterns; wide ranges vs. narrow bands; high vs. low frequency, etc. However, in all cases, such systems require higher efficiency and higher gains, with compatible penetration of human tissue. Jianli et al. [17] surveyed UWB antennas for medical applications and identified the following required features for medical imaging applications: ability to penetrate obstacles; higher precision range or multipath resolving capacity; low electromagnetic radiation (−41.3 dB); and lower energy consumption. A wireless interrogation system to acquire sensing data in the far field region of wireless communication was developed in [18] using reactive impedance surface ground based patch antennas. To provide the required features, a number of UWB antennas have been proposed: planar UWB antennas [19], square monopole antennas [20], square patch antennas [21], hook-shaped monopole antennas [22], tapered slot antennas [23], metamaterial-based UWB antennas [16,24], flexible coplanar waveguide fed (CPW-fed) fishtail antennas [25], semi-circular antennas [26], different types of Vivaldi antennas [27,28], and many more. A novel antenna miniaturization technique was introduced in [29], using reactive impedance surface as a substrate, which can be used as a perfect electric conductor as well as perfect magnetic conductor surface to enhance the bandwidth and radiation performance.

### 3.1. Operational Microwave Imaging System

The use of UWB antennas for microwave imaging began in the last decade. Researchers hypothesized that microwave imaging could be used to differentiate normal and tumorous tissues in terms of their electrical properties, permittivity, and conductivity. The dielectric properties of tumorous tissues are estimated to be six to ten times higher than those of normal tissues [30]. This occurs because tumorous tissues have a higher water content [31,32] than normal tissues or fat. UWB antennas are used as sensors for different sensing applications like temperature, moisture, crack, strain, microwave imaging etc. [33]. These antenna sensors are attractive because of their low cost, simple configuration, conformability, and capability of wirelessly transmit the acquired data to smart device [34]. 

To the best of our knowledge, UWB microwave technology was first used to detect early-stage breast tumors by Susan et al. [35]. The system used a single resistively loaded bowtie antenna as a sensor array. The researchers concluded that by combining existing equipment, the system could detect small cancerous tumors that were normally missed by X-ray mammography. The work was limited to simulation. An radio frequency identification (RFID) n sensor system was developed [36] using an ultra-wideband (UWB) antenna which modulated the amplitude of backscattering signal as a function of temperature. This made the UWB RFID reader low cost and portable.

**Figure 2:**
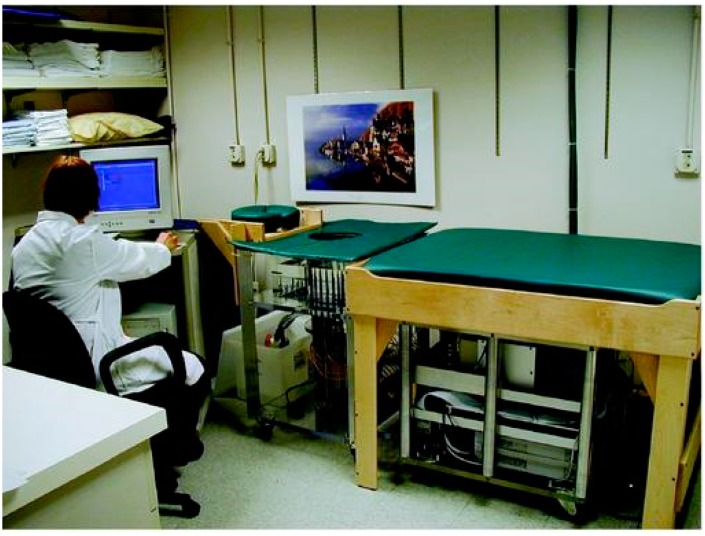
Microwave Imaging system at Dartmouth College [37].

A clinical prototype of near-field microwave imaging developed at Dartmouth college was presented by Meaney et al. [37]. The imaging setup is shown in Figure 2. Sixteen elements of a transceiving monopole antenna were used in the system within a frequency range of 300 to 1000 MHz. A tank was used as the coupling medium between the breast and the antenna. The antenna array was moved vertically and adjusted to chest level through a mechanical switch. Transmitter-receiver selection was performed by a microwave switch.

The development of a complete microwave imaging system was studied extensively by researchers with the advancement of antennas. The modalities of transceivers or sensors for microwave imaging are recorded in [38,39,40,41]. In [40], the transverse electromagnetic (TEM) Horn antenna was used to reduce complexity and did not require a matching liquid. However, in [39], Bourqui et al. augmented a Vivaldi antenna with a higher-dielectric material and director. This was done to focus the antenna’s power more effectively on the region of interest. The directive feature effectively increased scattered energy bounced from the tumorous region of the breast tissue. 

A comparison between a fork-fed wide slot antenna and cavity-backed stack patch antenna was performed at Bristol University [41] to identify the optimal antenna for microwave breast imaging. The wide slot antenna showed excellent performance over the frequency range. For the same frequency range, the wide slot antenna was three times smaller than the cavity-backed stack patch antenna, allowing for a more densely populated array. The comparison showed that the stacked patch antenna performance was satisfactory at angles close to bore-sight but that at wider angles, the performance degraded (timing was late and transmitted signals were distorted). 

Most of the antenna and imaging systems mentioned previously used matching liquid and plastic materials with properties close to those of real breast tissue. In [38], a UWB based microwave imaging system with experimental results was presented. A hemispherical array of stack patch antenna was used to detect breast cancer with a realistic breast phantom. Although mechanical scanning was not required, this was reported as the first use of real measured radar for breast cancer detection. However, there were some limitations related to resolution and cluster rejection. 

**Figure 3:**
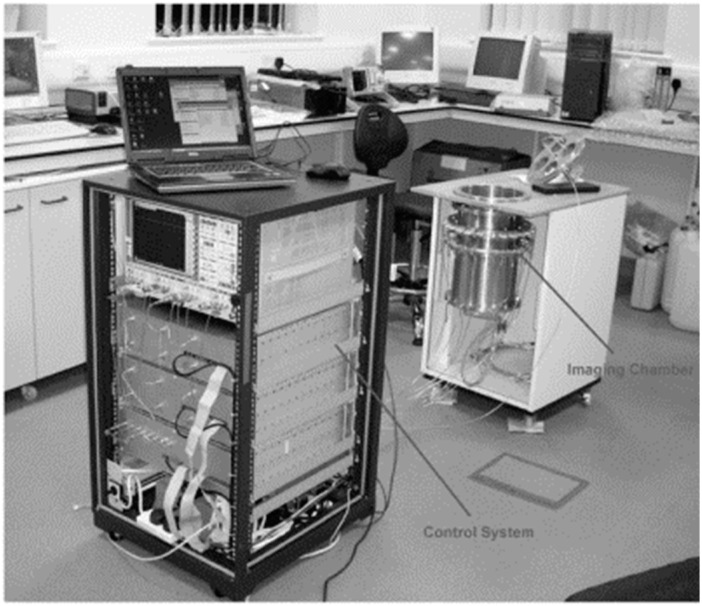
2D microwave tomography (MWT) system for soft tissue imaging and physiological activity detection [44].

Initial MIS measurements on animals were performed at the Carolinas Medical Center [42]. A 3D microwave imaging chamber was proposed, 120 cm in diameter and 135 cm in height. The system operated at 0.9 GHz. The time the complexity and mechanical scanning system were upgraded in [43] using a single transmitter and sixteen receiving antennas. The experiment was done to non-invasively assess myocardial tissues and detect infected tissues. A 2D configuration was developed to detect the functional and pathological activity of soft tissues [44]. The system was tested to detect the physiological activity and interventions of soft tissues. The system was built with a 2D imaging chamber comprising a metallic cylinder and 24 antennas, which were located equidistantly at the perimeter of the horizontal cross-section. The chamber was filled with mixtures of intralipid emulsion salt and alcohol with salt. The control unit transmits electromagnetic radiation to a single antenna and measures the electromagnetic field from all other 23 antennas. The system, which is shown in Figure 3, operated at a frequency range of 1 to 2.3 GHz. 

An iterative approach was proposed, in which the 2D Newton algorithm and 2D born method were used to present time differential images, as reported in [45]. 

Pilot clinical trials were carried out using suitable mechanical and electrical combinations [46]. Antipodal Vivaldi antennas were used to develop the monostatic system with mechanical scanning. A cylindrical tank was combined with a laser, with canola oil used as the coupling liquid. Consistent imaging results were obtained using delay and sum algorithms for image processing within a range of 50 MHz to 15 GHz. 

A hemispherical radome was used as a phantom and an antenna array of 16 elements was designed for a time domain radar system over a range of 2 to 4 GHz [47]. Porter et al. presented a clinical testing system capable of repetitive measurements and image reconstruction. The time domain radar system was designed using a pulse shaping circuit, reflector, directional coupler, and amplifier. The researchers used a time-equivalent oscilloscope and switching matrix to retrieve data from the receiver for image reconstruction. A total of 240 scanning signals from each antenna pair were obtained. The data were filtered and windowed. Finally, delay-multiply and sum algorithms were applied to obtain the 3D images. The proposed system reduced measurement uncertainty, noise, and general noise. The imaging algorithm was robust, and errors due to sensor and cable movements decreased [48,49,50,51]. However, the switching of several antennas is difficult. Additionally, in contrast with 3D scanning, part of the breast may not be scanned because of a lack of 360° coverage. 

The advancement of porter work at McGill University is presented in [52]. A 2D monopole system for breast tumor modeling is presented in [53,54]. A wideband antenna was used as a transceiver and low-cost off-the-shelf components replaced the costly vector network analyzer (VNA). The I-MUSIC algorithm was used for inversion in the bandwidth 0.5 to 3.0 GHz.

### 3.2. Tomographic Microwave Imaging System

**Figure 4:**
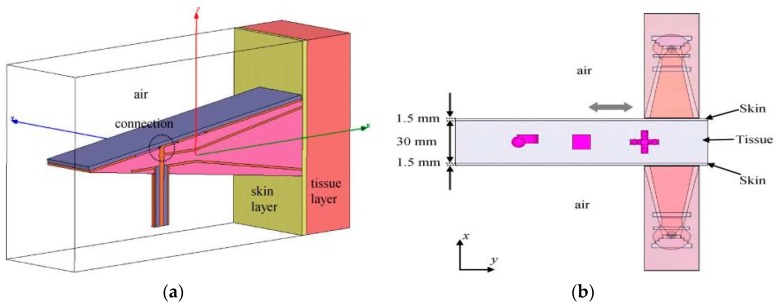
(**a**) Simulation setup of the antenna in a dielectric medium with microwave absorbing sheet on top surface (**b**) Imaging setup with compressed phantom and two antennas [55].

In 2011, Amineh et al. proposed a design and characterization of a UWB antenna based on a near-field imaging system, using raster scan [55] methods. The researchers presented both simulated and measured results using a homogeneous 3D phantom and heterogeneous model in which the antenna directly contacted the imaged body. The directive nature of the proposed UWB TEM horn antenna aided the scanning setup to obtain strong scattered signals inside the dielectric body. The simulation and measured antenna setup with an artificial phantom are shown in Figure 4. Two antennas were used, one as a transmitter and the other as a receiver. 2D scanning was performed by moving along the compressed breast in the opposite direction. To improve image quality, the images were de-blurred using a blind deconvolution algorithm [56]. Although the work was performed by scanning the phantom, the phantom that was used is not the same as a real breast and does not cover a 360° rotation angle. 

In [57], a mechanical scanner based 3D microwave imaging prototype was proposed using a rectangular tub filled with fluid as an imaging domain. To avoid mutual coupling problems, the system used two antennas: one as a transmitter and one as a receiver. By using a mechanical scanner, 3D experimental data were collected and an inversion algorithm was applied to reconstruct the nonlinear image. The inversion technique used two-step processing: the first step involved diagonal tensor approximation and used the born iterative method (BIM), while in the second step the biconjugate gradient first fourier transformation (FFT) was stabilized and a distorted BIM was applied to refine the images. The same experiment for both dielectric and metal spheres was also performed for layered media in [58]. Challenges associated with this system include the detection of tumors close to the skin and heterogeneous structures. 

**Figure 5:**
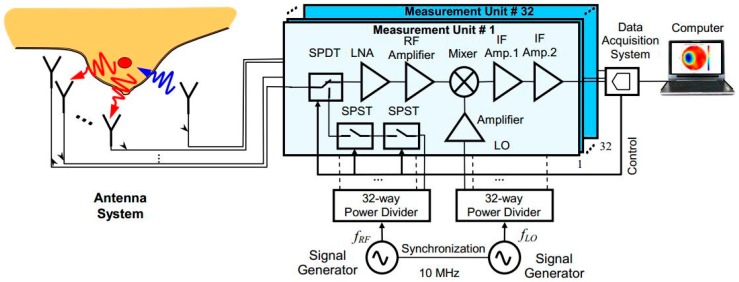
Schematic block diagram of microwave imaging [59].

**Figure 6:**
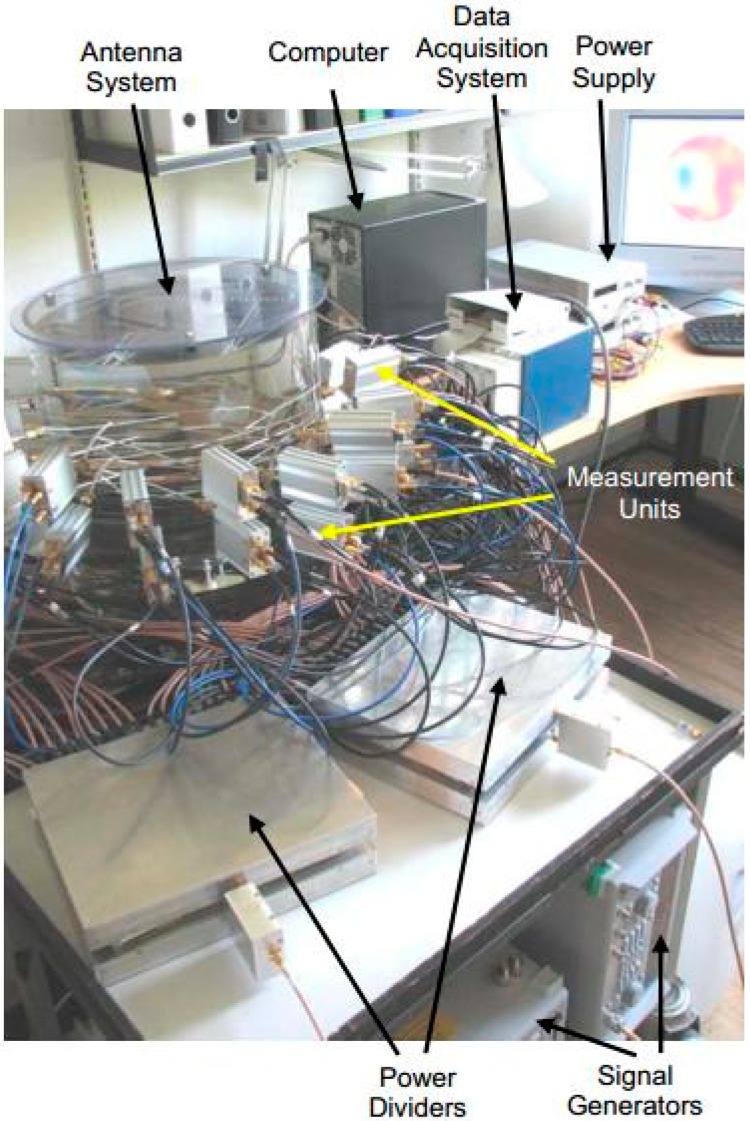
Photograph of the 3D imaging system [59].

A multichannel 3D architecture of microwave imaging was developed by Zhurbenko et al. [59] for non-invasive breast cancer detection. Sixteen pairs of identical transceiver channels were used to measure the vector electric field of the scattered signals in the 3D imaging domain. A diagram of the proposed system is shown in Figure 5 and a photograph of the system prototype is shown in Figure 6. The system was designed in a range of 0.3 GHz to 3 GHz and water-filled spheres of 20 mm and 40 mm in size were used as a target. From this lightweight measurement system, data were collected using a filter and analog-to-digital converter. These data were stored in the computer for use in the reconstruction algorithm. The nonlinear inverse scattering-based image reconstruction algorithm combined the moment forward solver method and Newton minimization algorithm. 3D images were obtained by consuming more than 100 min, with a reasonable size and shape of objects. 

### 3.3. Radar-Based UWB Microwave Imaging System

**Figure 7:**
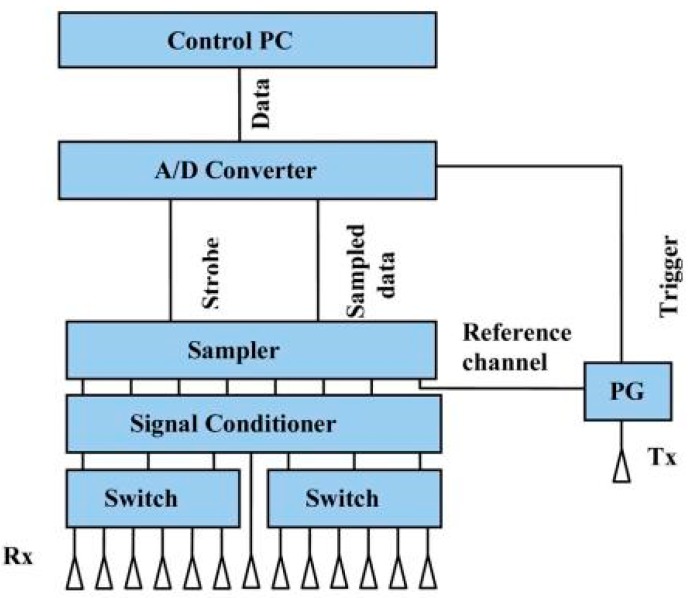
Block diagram of the array radar [60].

A UWB based near-field imaging system was developed by Yarovoy et al. [60]. An array-based sensor was designed for humanitarian demining, with a single transmitter and multiple receivers. A block diagram of the proposed array radar is shown in Figure 7. By using the mechanical scanner and electrical steering, the system produced 3D images of the subsurface. The bandwidth of the proposed system was 3.56 GHz which starts at 240 MHz. The diffraction stacking algorithm was combined with a synthetic aperture radar (SAR) algorithm to deliver 3D images of the target. Furthermore, Yarovoy et al. employed a 3D UWB based imaging system, versus the 2D systems of first generation.

**Figure 8:**
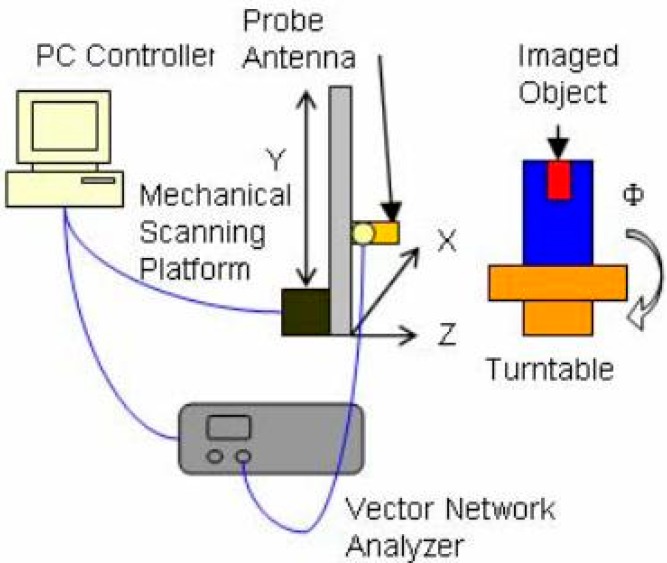
The system configuration of an ultra-wideband (UWB) radar [61].

A step frequency synthesis pulse technique based microwave imaging system for breast cancer detection was presented in [61]. The system used a Φ-Y circular scanning platform with a turntable resolution of 22.5°. A cylindrical container with low-dielectric vegetable oil and a small target with a high dielectric constant was scanned with a tapered slot UWB antenna having a range of 3.1 to 10.6 GHz. The reflection coefficients of the frequency domain were measured and converted to the time domain using inverse first fourier transformation (IFFT). The data were processed in a PC and an image was created of the breast phantom with the distinct target color. The configuration of the radar system is shown in Figure 8. This system overcame several problems associated with first generation MISs.

A pre-clinical prototype was investigated by Flores et al. in [62]. A single-element Vivaldi antenna based system was designed to study the cylindrical dielectric targets. The plexiglass tank used as a phantom was filled with canola oil to measure reflection coefficient (S_11_) and to improve the quantitative dielectric images using datasets of rotating phantom circular scan geometry. 

**Figure 9:**
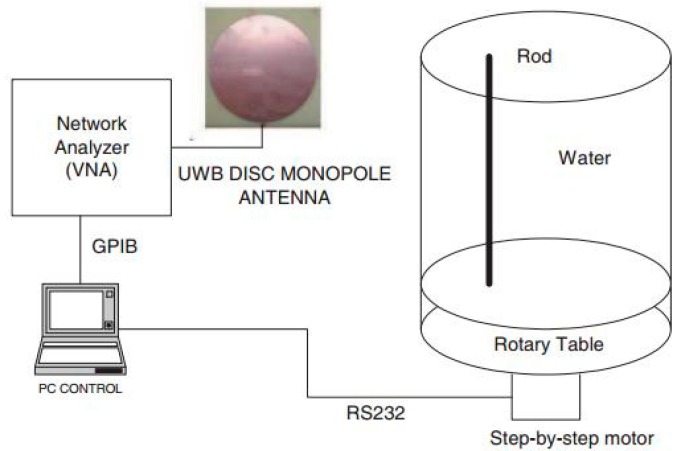
Experimental setup of working prototype [63].

The feasibility of detecting tumors using UWB signals with a finite-difference time-domain (FDTD) method is presented in [63]. In the experimental setup, a UWB antenna ranging from 0.5 to 3 GHz was connected to a vector network analyzer, as shown in Figure 9. The water filling the cylindrical glass tank was used as a phantom, while an immersed low-permittivity rod was used to model a tumor. The losses and permittivity of water are much higher than those of both air and real breast tissue. This creates higher attenuation and reflection at the air-skin interface, a characteristic that was employed in the experimental method. The researchers investigated changes in the sensitivity of breast and skin surface contours. Mohammed et al. at the University of Queensland presented a UWB planner of 12 tapered slot antennas (TSAs) for microwave imaging applications [64]. A suitable platform was designed combining a vector network analyzer (VNA), single-pole through (SP6T) switch, breast phantom, and coupling liquid. The water cylinder, conductor, and dielectric were flooded in a plastic container filled with canola oil. A trust region framework was used to observe the mutual coupling and the system was tested for breast imaging applications. The system presented a preparation test platform but did not provide any real image and scattering signal processing, because it was limited to the mutual coupling between each antenna pair.

Zhang et al. [65] presented a simulation experiment system for breast tumor detection using compact UWB Vivaldi antennas. A commercially available breast phantom and cooking oil were used as the matching liquid in the system. A sampling oscilloscope was used to receive the transmitted pulse. The reflection data were collected using a background subtraction method from the hardened liquid embedded within the metallic ball. The breast cancer tumor was detected using a confocal microwave imaging (CMI) algorithm.

Globally, a number of MISs have been developed for breast tumor detection. Some of them have used 2D arrangements, while a few of have used 3D arrangements with distinct types of antennas. Most of the systems use UWB antennas, based on several different prototypes. The MISs are summarized in Table 2. Most of the systems measured non-biological phantoms, not maintaining the original tissues’ and tumors’ electrical properties.

## 4. Challenges of Microwave Imaging (MWI) and Future Development

Over the last three decades, researchers have investigated microwave imaging, with the goal of developing high performance, low-cost tools as alternatives to existing medical imaging systems for breast tumor detection. By using transmitting and receiving antennas, penetrating electromagnetic waves are used to reconstruct the dielectric properties and shapes of simple breast models. There are some major challenges associated with applying microwave imaging to practical real time breast imaging. The development of a program for inverse scattering [68,69] and image reconstruction with a small sensor is the first challenge facing MWI. Secondly, in most of the experimental systems, the breast phantoms used were simple and homogeneous, unlike real human breast tissue. Thirdly, the perfect operating frequency range for microwave imaging is still under investigation. The differences between healthy and tumorous cells much smaller than appreciated, making image construction difficult. One potential solution to these challenges may be combining hybrid imaging and scattering programs with commercial electromagnetic (EM) simulators. A number of previous investigations have increased the number of antennas in order to solve the spatial resolution problem [70]. This approach made the MWI system more complex, and decreased the detection accuracy because of mutual coupling between antennas (at a higher cost). In one very recent investigation, a multiple input and multiple output (MIMO) technique was used to reduce system complexity [71]. 

## 5. Conclusions

In this paper, we presented up-to-date reviews of UWB antenna sensor features and current UWB applications in the field of breast imaging. A number of MWI approaches were reviewed. We broadly discussed the limitations of existing detection techniques, including the open challenges of MWI and probable solutions. From this discussion, we can conclude that UWB-based MWI has improved dramatically. Within the time being and have a major motivation in earlier breast tumor detection by relating the dielectric properties of human tissues with biological circumstances. Despite certain limitations, MWI techniques have several promising characteristics and because of these, MWI is likely to become a successful clinical complement to conventional medical imaging tools. MWI techniques must be developed further; commercial companies may help develop a well-established MWI system for application in clinical environments.

## Figures and Tables

**Table 1:** Comparison of Diagnostic Performance of different breast tumour detection techniques [14].

Modality	Sensitivity	Specificity	Positive Predictive Value	Accuracy	Advantages	Limitations
Mammography	67.8% (120/177)	75% (61/81)	85.7% (120/140)	70.2% (181/258)	Low cost	False positive and negative diagnoses
Mammography and clinical examination	77.4% (137/177)	72% (58/81)	58.6% (137/160)	75.6% (195/258)	Low cost	Lower accuracy
Clinical examination	50.3% (89/177)	92% (75/81)	94% (89/95)	63.6% (164/258)	Simple and easy process	Small tumor cannot detect
Ultrasound	83% (147/177)	34% (28/81)	73.5% (147/200)	67.8% (175/258)	Better than X-ray	Difficult to detect deep-lying or solid tumor
Mammography and ultra sound	91.5% (162/177)	23% (19/81)	72.3% (162/224)	70.2% (181/258)	Cost effective	Exists unwanted compression
Mammography ultrasound and clinical examination	93.2% (165/177)	22% (18/81)	72.4% (165/228)	70.9% (183/258)	Good candidate for detection	Complex signal processing
MRI	94.4% (167/177)	26% (21/81)	73.6% (167/227)	72.9% (188/258)	Provide high resolution images	Higher cost and time-consuming process
Mammography, clinical examination, and MRI	99.4% (176/177)	7% (6/81)	70.1% (176/251)	70.5% (182/258)	Best solution ever found	Complex procedure, higher cost and time-consuming process

**Table 2:** Summarized Table of UWB imaging.

Origin	Imaging Domain	Antenna Configuration	Targets	Results
Dartmouth College [37,66]	Cylindrical tank (agar gel, corn syrup, water mixer)	16 antennas Mechanical scanning	Detecting malignant tumors	2D and 3D images
University of Bristol [38,67]	Acrylonitrile butadiene styrene plastic half sphere	UWB radar	Measure the symptomatic patients	First real breast phantom but limitations in terms of resolution and clutter rejection
Carolinas medical center [44,45]	Metallic tank 21.5 cm in diameter	24 waveguide antennas	Detection of physiological activity of soft tissues	2D and 3D tomographic images of swine torso obtained
University of Calgary [46]	Tank with canola oil	Single balanced antipodal antenna with mechanical scanning	Pilot clinical experiment	Consistent imaging results
University of Manitoba [62]	Plexiglass tank with canola oil	Single Vivaldi antenna	Pre-clinical UWB prototype	Improvements to quantitative dielectric image
University Rovira [63]	Water filled Cylindrical glass tank	UWB disc monopole antenna	Working prototype for microwave imaging	Tumor position was detected
University of Queensland [64]	Plastic container filled with canola oil	12 UWB antennas	UWB biomedical imaging	Mutual coupling and fidelity
McGill University [47]	Hemispherical ceramic (Al_2_O_3_) radome	16 elements antenna array	Clinical testing	First study of microwave time domain with actual volunteers
Politecnico di Torino [53]	Metallic Cylinder	Monopole 8 element antenna array	Design and construction of imaging prototype	2D imaging at MiMed cost meeting
Duke University [57,58]	Rectangular tub filled with fluid	Two dipole antennas	3D imaging system prototype	5 mm diameter dielectric objects detected
Toyohashi University of Technology [65]	Rectangular tub with cooking oil	UWB Vivaldi antennas	Breast cancer tumor detection	9 mm metallic ball detected
Technical University of Denmark [59]	Water filled spheres	32 monopole antennas	20 to 40 mm target objects detection	3D images are obtained with consuming more than 100 min
McMaster University [55]	Glycerin based flat artificial phantom	2 TEM horn antenna	3D model and phantom analysis where antenna directly contacted imaged body	Image de-blurred using blind deconvolution algorithm
